# Silk Fibroin as Adjuvant in the Fabrication of Mechanically Stable Fibrin Biocomposites

**DOI:** 10.3390/polym14112251

**Published:** 2022-05-31

**Authors:** Ikram El Maachi, Stavroula Kyriakou, Stephan Rütten, Alexander Kopp, Marius Köpf, Stefan Jockenhoevel, Alicia Fernández-Colino

**Affiliations:** 1 Department of Biohybrid & Medical Textiles (BioTex), AME-Institute of Applied Medical Engineering, Helmholtz Institute, RWTH Aachen University, D-52074 Aachen, Germany; el-maachi@ame.rwth-aachen.de (I.E.M.); kyriakou@ame.rwth-aachen.de (S.K.); 2 Electron Microscopy Facility, Uniklinik RWTH Aachen, D-52074 Aachen, Germany; sruetten@ukaachen.de; 3 Fibrothelium GmbH, D-52068 Aachen, Germany; alexander.kopp@fibrothelium.com (A.K.); marius.koepf@fibrothelium.com (M.K.); 4 AMIBM-Aachen-Maastricht-Institute for Biobased Materials, Faculty of Science and Engineering, Brightlands Chemelot Campus, Maastricht University, 6167 RD Geleen, The Netherlands

**Keywords:** protein-based polymers, scaffolds, cell-adhesion, bioprocessing, mechanical stability, tissue engineering

## Abstract

Fibrin is a very attractive material for the development of tissue-engineered scaffolds due to its exceptional bioactivity, versatility in the fabrication, affinity to cell mediators; and the possibility to isolate it from blood plasma, making it autologous. However, fibrin application is greatly limited due to its low mechanical properties, fast degradation, and strong contraction in the presence of cells. In this study, we present a new strategy to overcome these drawbacks by combining it with another natural polymer: silk fibroin. Specifically, we fabricated biocomposites of fibrin (5 mg/mL) and silk fibroin (0.1, 0.5 and 1% *w*/*w*) by using a dual injection system, followed by ethanol annealing. The shear elastic modulus increased from 23 ± 5 Pa from fibrin alone, to 67 ± 22 Pa for fibrin/silk fibroin 0.1%, 241 ± 67 Pa for fibrin/silk fibroin 0.5% and 456 ± 32 Pa for fibrin/silk fibroin 1%. After culturing for 27 days with strong contractile cells (primary human arterial smooth muscle cells), fibrin/silk fibroin 0.5% and fibrin/silk fibroin 1% featured minimal cell-mediated contraction (ca. 15 and 5% respectively) in contrast with the large surface loss of the pure fibrin scaffolds (ca. 95%). Additionally, the composites enabled the formation of a proper endothelial cell layer after culturing with human primary endothelial cells under standard culture conditions. Overall, the fibrin/silk fibroin composites, manufactured within this study by a simple and scalable biofabrication approach, offer a promising avenue to boost the applicability of fibrin in tissue engineering.

## 1. Introduction

Tissue engineering (TE) aims to obtain biological substitutes that can restore the functionality of native tissue in vivo. This discipline of biomedical engineering pursues the development of constructs able to interact with cells and to evolve to a native-like tissue, either in vitro (classical TE approach) or in vivo (in situ TE approach). Therefore, the development of advanced materials, capable of mimicking the native extracellular matrix (ECM) and/or promote new tissue formation, is pivotal for the progress of TE [1].

In practice, a single material can hardly ever meet all the requirements needed for full functionality as the target tissue. Indeed, the outstanding performance of many natural systems (including the native ECM) [2,3,4] responds to a unique combination of materials with complementary or even contradictory properties. This composite strategy, followed by nature [5], should be further exploited in materials science in order to enable synergy between the different components and to consequently match the requirements for tissue regeneration [6].

Fibrin is described in many studies as an ideal biomaterial for many biomedical applications, owing to its bioactivity, versatility and processability [7,8]. Indeed, the use of fibrin as a biomaterial has been expanded from tissue sealants [9] to a myriad of TE and drug delivery applications [10], including skin wound healing [11], reconstructive maxillofacial and dental surgeries [12], eye surgery [13], cartilage regeneration [14], orthopaedic surgery [15] and cardiovascular implant development [16,17], among others. Fibrin can be isolated directly from the patients’ plasma and processed into a scaffold [18]. Therefore, a scaffold made of the patient fibrin and cultivated with the same patient cells, will provide a total autologous construct, which removes any immunological or by-product issues. While blood extraction from the patient may not always be possible (e.g., due to severe injuries or emergencies), fibrin from non-autologous sources have demonstrated great potential. Indeed, commercial fibrin sealants are homologous, i.e., they contain human blood components from either pooled plasma or individual units of plasma. In order to reduce the production costs and increase the availability, heterologous forms of fibrin are emerging as a promising approach [19], and indeed some formulations have reach clinical trials [20].

Fibrin displays a prominent bioactivity, due to the presence of the arginine-glycine-aspartic (RGD) sequence in the fibrin network [21]. The RGD motifs together with the cell integrins, create a recognition system for many cell types in the human body, and additionally, they support the attachment of growth factors and essential molecules to the fibrin substrate [22,23]. Additionally, fibrin degradation products are also acknowledged as important modulators of wound healing [24,25].

Despite these advantages, the potential of fibrin in TE is mainly limited by mainly two factors: (i) cell-mediated scaffold contraction, and (ii) insufficient mechanical strength to withstand physiological forces, e.g., hemodynamic ones [26]. Therefore, the identification of a biomaterial able of enhancing the mechanical behavior of fibrin, while still preserving its excellent biological properties, can make an outstanding contribution to the field of TE. In this regard, we hypothesize that blending fibrin with silk fibroin, as natural polymer with superior mechanical strength, could be a promising approach. Silk fibroin is an extraordinary versatile natural material that can be used in a plethora of biomedical applications [27]. Indeed, silk fibroin has been clinically used for decades as a suture line, and there are many examples of silk fibroin-based medical devices approved by the FDA [28,29,30].

Currently, only a few studies have explored the blending of silk fibroin with fibrin. One study combined fibrin gels with silk fibroin by coating the silk fibroin’s porous scaffolds after seeding the cells in order to support the formation of capillary structures [30]. In that approach, the fibrin was used merely as a coating for the silk fibroin scaffold, and therefore, it was not fully integrated in the whole scaffold structure. Another study combined fibrin with a silk fibroin network through an interpenetrating polymeric network (IPN) architecture [31]. The IPN architecture was synthetized with a double enzymatic method, which consisted of the use of thrombin enzyme for the gelation of the fibrin, and horseradish peroxide for the silk fibroin gelation. The study showed promising results regarding the fibrin and silk fibroin blend. However, the method adopted in that study carries numerous disadvantages. The double enzymatic method is an expensive approach, which therefore limits the scalability and consequently the industrialization of these scaffolds A further limitation is given by the slow gelation using the enzymatic treatment. Although a faster gelation of the silk fibroin can be achieved by increasing the concentration of the horseradish peroxide, a high concentration of this enzyme may be cytotoxic [32]. Additionally, the cellular study was limited to a single cellular type, fibroblasts, which are well-known to be more resilient than other cell types (e.g., endothelial cells).

In this study, we propose a strategy to fabricate composite fibrin/silk fibroin scaffolds in simple and reproducible way. Our motivation was to investigate if a proper balance of mechanical properties and cell adhesion could be achieved and consequently, if the composite scaffolds could exceed the fibrin ones for their application in TE.

## 2. Materials and Methods

### 2.1. Fibrin and Silk Fibroin Preparation

Lyophilized human fibrinogen (Human plasma, Calbiochem, San Diego, CA, USA) was dissolved in purified water (Sartorius, Goettingen, Germany) and dialyzed against Tris-buffered saline (TBS) overnight using Spectra/Por 1 tubing (Fisher Scientific, Schwerte, Germany) with a molecular weight cut-off of 6000–8000 Da. Fibrinogen concentration following sterile filtration was determined by measuring absorbance at 280 nm using a M200 spectrophotometer (Tecan Group Ltd., Männedorf, Switzerland). Fibrinogen was diluted in TBS to a final concentration of 10 mg/mL and 20 mg/mL. The thrombin solution was made by mixing 15% (*v*/*v*) of thrombin (bovine plasma, Sigma Aldrich, Saint Louis, MO, USA) at 40 U/mL, 15% (*v*/*v*) of Calcium chloride (CaCl_2_, Sigma-Aldrich, Saint Louis, MO, USA) at 50 mM and 70% (*v*/*v*) of TBS. The TBS buffer was prepared by dissolving Tris HCl, Tris base, NaCl and KCl in purified water and adjusting the pH to 7.4.

Silk fibroin solution was provided by Fibrothelium GmbH and produced according to the PureSilk^®^ technology established by the company. Upon receipt, it was diluted in ultrapure water at a concentration of 2%, 1% and 0.2% (*w*/*v*).

### 2.2. Fabrication of Composite Scaffolds

A double dual injection system was adopted to fabricate the composite scaffolds. The system consisted of syringes containing the fibrinogen, thrombin and silk fibroin solutions placed in a Duploject double syringe holders (Tisseel, Baxter, Deerfield, IL, USA), fitted into the dual injectors and connected to a 3-way connector. To combine the solutions homogenously, a mixing nozzle (DMG, Hamburg, Germany) was inserted at the end of the system.

The solutions were injected in the mold with the desired shape and left for 20 min to allow the complete polymerization of the fibrin. The samples were then immersed in 100% ethanol (Sigma-Aldrich, Saint Louis, MO, USA) for 2 h in order to promote the β-sheets’ formation and, therefore, the crosslinking of silk fibroin. Afterwards, the samples were washed three times with PBS (Thermo Fisher, Waltham, MA, USA) to avoid any ethanol residues. The ratio and the nomenclature of the conditions and controls used in the experiments are stated in Table 1.

### 2.3. Scanning Electron Microscopy (SEM)

SEM (ESEM XL 30 FEG, Eindhoven, Netherlands) was used to investigate the surface and the internal morphology of the composite scaffolds.

The samples were subjected to freeze-drying in order to make them suitable for SEM visualization. Specifically, the applied process involved three steps: (i) immersion in liquid nitrogen for a few seconds, (ii) fracturing (while immersed) to visualize later on the internal structure and (iii) lyophilization. The scaffolds were lyophilized in an Alpha 2-4-LSCplus freeze dryer (Christ, Osterode am Harz, Germany) at −20 °C for the first 12 h, and at 20 °C for another 12 h for the final drying.

After lyophilization, the scaffolds were placed in a wellplate, vacuumed and moved to the Electron Microscopy Facility (Institute of Pathology, RWTH Aachen University Hospital, Aachen, Germany). The samples were carefully mounted on an aluminium stub and sputter-coated with 12.5 nm Pd-Au alloy (EM SCD500, Leica, Wentzler, Germany), and then visualized in high vacuum mode.

### 2.4. Rheological Characterization

For the rheological characterization, scaffolds were fabricated with the composition as stated in Table 1 and with a diameter of 20 mm. To observe the influence of the annealing of silk fibroin within the composites, the composite scaffolds (i.e., Fib5-Silk 0.1%, Fib-Silk 0.5% and Fib5-Silk 1%) without ethanol treatment were also measured.

The scaffolds were measured in the Malvern Kinexus ultraplus rheometer (Malvern, Worcestershire, UK), using a geometry of 20 mm in diameter. The plate was heated at 37 °C and a water trap was placed to avoid the dehydration of the samples. The gap was adjusted to a minimum normal force of 0.01 N to avoid the slippage of the sample. A strain sweep oscillation test was performed on the scaffolds to identify the linear viscoelastic regions (LVE) of the samples. At a fixed frequency of 1 Hz, the storage modulus (G′) and the loss modulus (G′′) were measured as a function of the shear strain with an amplitude ranging from 0.01 to 100%. Five samples were measured per condition, and the data were expressed as mean and the corresponding standard deviation (SD) of the five replicas.

### 2.5. Cell Culture Study

#### 2.5.1. Culture of Human Umbilical Vein Endothelial Cells (HUVECs) and Human Umbilical Arterial Smooth Muscle Cells (HUASMCs)

Endothelial cells (HUVECs) and smooth muscle cells (HUASMCs) were isolated from human umbilical cord [33], as previously described [34]. Human umbilical cords were obtained after written consent at the University Hospital Aachen, Aachen, Germany, and were provided by the RWTH Aachen University Centralized Biomaterial Bank (cBMB) according to its regulations, following RWTH Aachen University, Medical Faculty Ethics Committee approval (cBMB project number 323). HUVECs were cultured on 2% gelatine (Sigma-Aldrich, Saint Louis, MO, USA) coated flasks in an incubator at 37 °C with 5% CO_2_ and fed with EGM (PromoCell, Heidelberg, Germany). The following supplements were added to the media: basic Fibroblast Growth Factor, Insulin-like Growth Factor, Vascular Endothelial Growth Factor 165, Ascorbic Acid, Heparin and Hydrocortisone. Conversely, HUASMCs were cultured in cell culture flasks with Dulbecco′s Modified Eagle’s Medium (DMEM, Thermo Fisher, Waltham, MA, USA) supplemented with FCS and 1% (*v*/*v*) ABM, in an incubator of 37 °C and 5% CO_2_.

The media of the two cell types were changed every two days and at confluence the cells were trypsinized and centrifuged at 500 g for 5 min. The resulting cell pellets were suspended within the respective media at the desired cell concentration.

#### 2.5.2. Seeding of HUVECs and Evaluation of Cell Adhesion

The composite scaffolds of silk fibroin and fibrin were produced as described in Section 2.2, with three replicas for each condition. Additionally, scaffolds containing only fibrin or containing only silk fibroin were included in the assay as controls.

**Figure 1:**
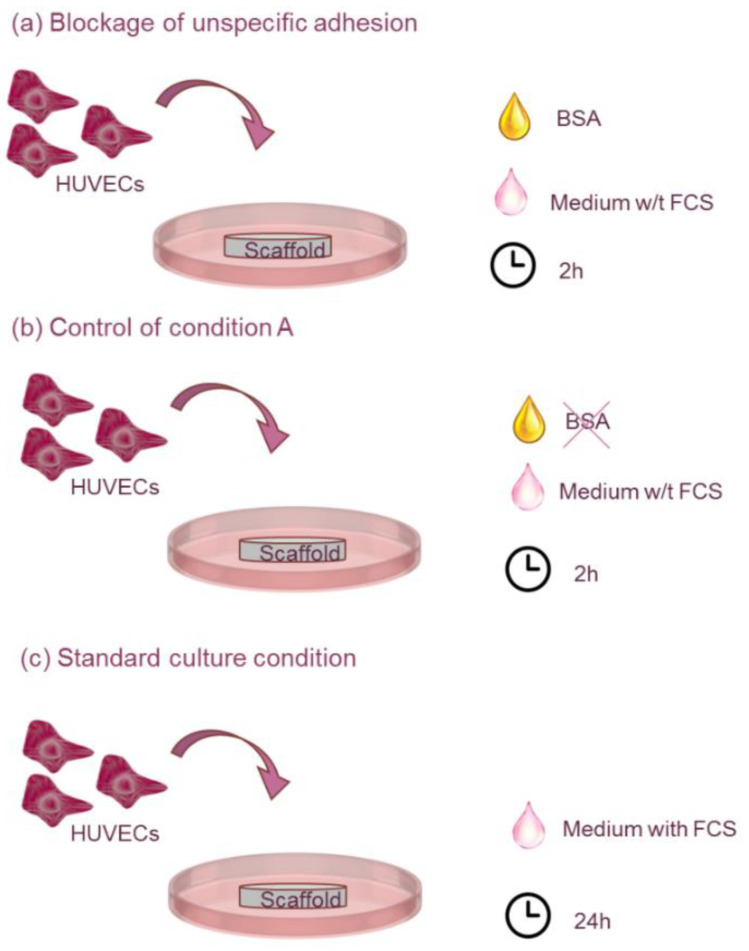
Schematic representation of the experimental groups assessed for adhesion of human umbilical vein endothelia cells (HUVECs) to the composite scaffolds. (**a**) HUVECs suspended in media without FCS and seeded on BSA-treated scaffolds. (**b**) HUVECs suspended in media without FCS and seeded on scaffolds (non BSA-treated). (**c**) HUVECs suspended in media with FCS and seeded on scaffolds without BSA pre-treatment.

Two experimental groups were established, i.e., (i) scaffolds pre-treated with Bovine Serum Albumin (BSA, Sigma-Aldrich, Saint Louis, MO, USA) at 10 mg/mL in PBS for 2 h and rinsed three times with PBS, and (ii) scaffolds without BSA treatment (Figure 1a,b). BSA is as a blocking agent, known to impede any unspecific adhesion to the scaffolds [35]. In both cases, HUVECs suspended in media without FCS were added to the samples at a concentration of 1.26 × 10^5^ cells/cm^2^, and incubated at 37 °C with 5% CO_2_ for 2 h.

Additionally, a third set of scaffolds (non-treated with BSA) was cultured for 24 h with cells suspended in FCS-containing media in order to assess the capability of the scaffolds to support the cell attachment under standard culture conditions (Figure 1c). Each experimental group contained 3 replicas of each type of scaffolds.

#### 2.5.3. Seeding of HUASMCs and Evaluation of Scaffold Contraction

Scaffolds with a disk-shape of 2 cm diameter and 2 mm thickness were prepared as described in Section 2.2, transferred to a 12-wellplate and imaged with a digital camera in order to accurately evaluate the surface at time 0 (before seeding). Afterwards, HUASMC suspension at 1 million cells/mL was seeded on the different scaffolds. Additionally, fibrin scaffolds at 10 mg/mL were included in these experiments, in order to determine whether an increase in the material concentration makes an impact in the stability or contraction of the scaffold. Four replicas of each condition were seeded with cells, and three additional ones were kept unseeded in order to discern whether the contraction of the scaffolds was cell-mediated.

After 24 h of culture, one replica of each condition of the seeded scaffolds was fixed with paraformaldehyde (Carl Roth, Germany) (Section 2.5.4), stained with phalloidin/DAPI and visualized in the confocal microscope. The rest of the scaffolds (*n* = 3 per condition) were cultured for 27 days. Every second day, the medium was changed, and pictures of the scaffolds were acquired in order to monitor changes in the surface, if any. To this end, the images were collected, and then analysed with the software ImageJ (National Institutes of Health, Bethesda, MD, USA).

#### 2.5.4. Fixation and Staining of the Cell-Seeded Scaffolds

After the respective incubation times, the cell-seeded scaffolds were washed one time with PBS and then fixated in 4% paraformaldehyde (methanol-free) diluted in PBS, for 1 h at room temperature. Subsequently, they were rinsed in PBS three times and stored in PBS containing antibiotics/antimycotic (ABM) (Pan Biotech, Aidenbach, Germany) at 4 °C, until staining.

The samples, prior to staining, were permeabilized with the washing buffer (PBS with 0.1% Triton X-100, Sigma-Aldrich, Saint Louis, MO, USA) for 5 min and rinsed three times in PBS. Phalloidin-ifluor 488 conjugate (Cayman chemical, Ann Arbor, MI, USA) with a 1000× dilution in 1% (*w*/*v*) BSA in PBS, was added to the samples to stain the actin filaments in the cell’s cytoskeleton, with an incubation time of 90 min at room temperature. The samples were rinsed three times in PBS and the nuclei were stained with DAPI (Carl Roth, Karlsruhe, Germany) for 15 min and then washed again in PBS for three times.

#### 2.5.5. Confocal Microscopy Evaluation

The samples were imaged with an inverted Zeiss LSM710 laser scanning confocal commercial microscope at the Confocal Microscopy Facility (IZKF, RWTH Aachen University Hospital, Aachen, Germany). The images were taken with both EC Plan-Neofluar 10× and Plan Apochromat 20× objectives. Images were acquired sequentially by line scanning unidirectionally and saved as czi files. For the visualization of DAPI staining, the samples were excited with a 405 nm laser line, and emission was collected at 410–495 nm, while, for the visualization of the actin-iFluor 488-staining, the excitation was performed with a 488 nm laser line, and emission was collected at 495–630 nm. The software Zen black 2012 (Zeiss, Jena, Germany) was used for image acquisition and ImageJ was used to count the number of the cells.

### 2.6. Fabrication of Tubular Scaffolds

The silk fibroin and fibrin components were bioprocessed in a similar way, as described in Section 2.2, but this time, they were injected into a custom-made tubular mold. Specifically, the mold had a 1 mm of wall thickness, a 5 cm of length and a diameter of 1.2 cm.

### 2.7. Burst Strength

The burst strength was measured with an in-house device, which consists of a peristatic pump (IPC Ismatec, IDEX Health & Science, Carlsbad, CA, USA) that pumps PBS with a rate of 7.5 mL/min inside of a chamber where the samples are positioned. The pressure sensor (Jumo Midas pressure transmitter, JUMO, Fulda, Germany) attached to the chamber, measures the pressure that the PBS applies on the sample. The peak of the pressure measured, at which the samples rupture, is the burst pressure. Since the burst pressure is directly correlated to the thickness of the sample, the scaffolds were prepared in a way to attain a reproducible and equal thickness. i.e., as stated in Section 2.6. The tubular samples were cut longitudinally, and the resulting sheets were laid down on the device chamber. Three measurements were taken for each condition, and the peak of the pressure was noted. The recorded data are expressed as mean ± SD.

### 2.8. Statistical Analysis

All data were represented as mean ± SD from at least three experiments. Statistical analysis of the rheological measurements and the HUVECs adhesion tests results were performed with two-way analysis of variance (ANOVA) with a Holm- Šídák test. A one-way ANOVA (Holm-Šídák) was employed in the case of the burst pressure results data. A *p*-value < 0.05 was considered statistically significant.

## 3. Results

### 3.1. Fabrication of the Scaffolds

**Figure 2:**
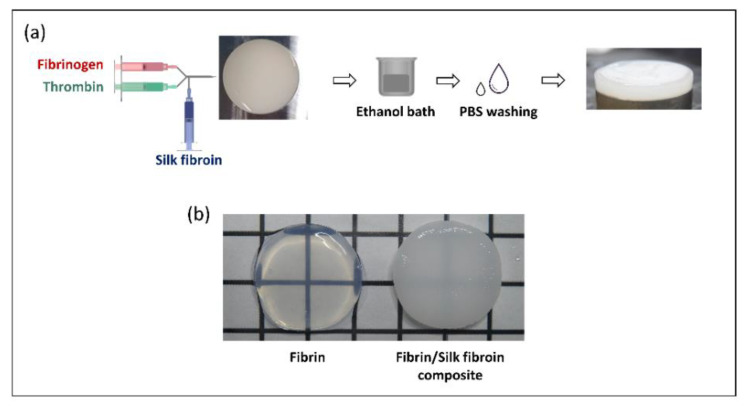
(**a**) Schematic representation of the fabrication process of the composite scaffolds. (**b**) Macroscopic picture of a pure fibrin scaffold and a fibrin/silk fibroin scaffold.

Fibrin/silk fibroin composites with different silk fibroin concentrations (0.1%, 0.5% and 1% *w*/*w*) were successfully fabricated by using a dual double injector system (Figure 2a). By increasing the silk fibroin content, a better processing and less deformation on the samples were observed, with an associated change in colour, from an almost transparent behavior of the pure fibrin scaffolds to a whiter (and less transparent) colour on the composites (i.e., fibrin + silk fibroin) (Figure 2b).

**Figure 3:**
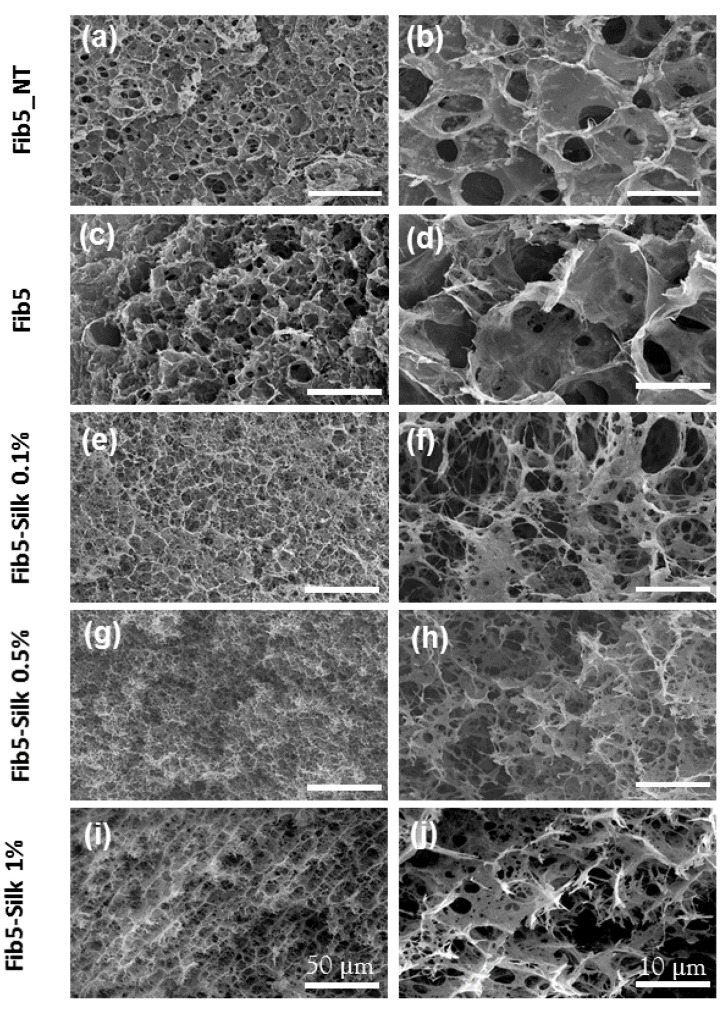
Scanning electron microscopy (SEM) images of fibrin and fibrin/silk fibroin scaffolds. (**a**,**b**) Fibrin scaffold at 5 mg/mL (without ethanol treatment). (**c**,**d**) Fibrin at 5 mg/mL with ethanol treatment. (**e**,**f**) Composite of fibrin (5 mg/mL) and silk fibroin (0.1% *w/w*). (**g**,**h**) Composite of fibrin (5 mg/mL) and silk fibroin (0.5% *w*/*w*). (**i**,**j**) Composite of fibrin (5 mg/mL) and silk fibroin (1% *w/w*).

The surface and the inner morphology of the samples were characterized by SEM. All the scaffolds presented an open porous structure (Figure 3), and the presence of the silk fibroin in the composites did not hamper the consecution of a sponge-like internal structure.

### 3.2. Rheological Characterization

Rheological experiments were carried out to evaluate the mechanical behavior of the composite scaffolds and to elucidate (i) whether the incorporation of silk fibroin has an impact on the mechanical performance of the scaffolds and (ii) whether the silk fibroin annealing state affects the mechanical behavior of such composites.

**Figure 4:**
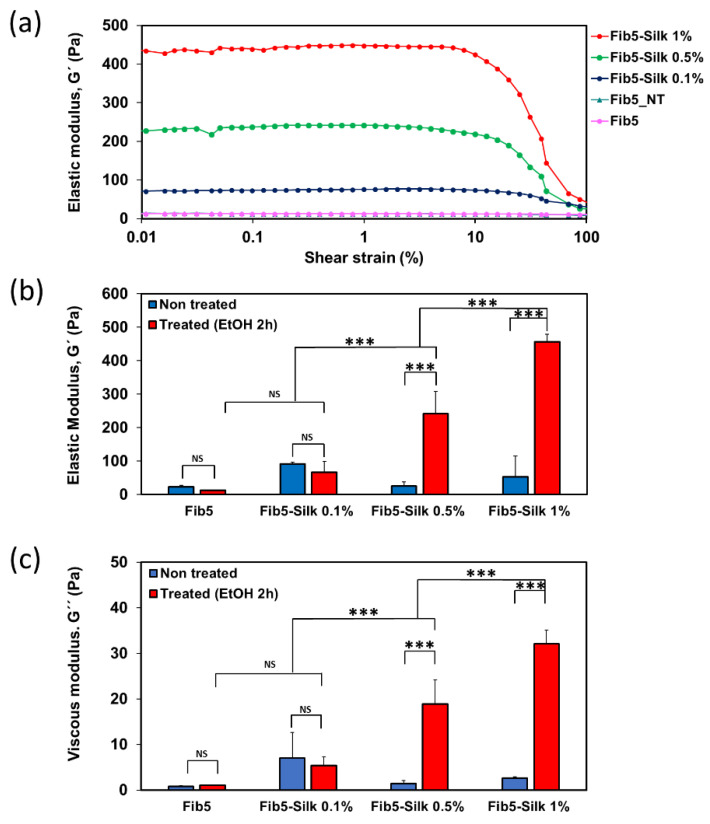
Rheological characterization of fibrin and fibrin/ silk fibroin scaffolds. (**a**) Dependence of the elastic moduli with the shear strain at 1 Hz. A representative replica for each scaffold is shown. (**b**) G′ and (**c**) G′′ measurements at 0.4% of shear strain and 1 Hz of frequency. Data is expressed as mean ± SD (*** *p* < 0.001, NS: not significant).

All the samples featured a stable elastic modulus (G′) regardless of the shear strain for the range between 0.01% and 8% (Figure 4a), which was therefore stablished as the linear viscoelastic region (LVR) of the scaffolds. For shear strain values higher than 8%, the elastic moduli experienced an evident decrease, especially evident for those samples containing higher percentages of silk fibroin.

For the non-treated scaffolds (i.e., before the crosslinking of the silk fibroin with ethanol; Figure 4b-blue columns), no correlation was observed between the amount of silk fibroin in the composite and the mechanical properties, with all the samples featuring moduli below 100 Pa (Figure 4b-blue columns). This drastically changed once the scaffolds were treated with ethanol (Figure 4b-red columns). In that case, the elastic moduli experienced a clear increase by increasing the silk fibroin content in the scaffold (*p* < 0.001), from a modulus of 67 ± 22 Pa for Fib5-Silk 0.1%, to 241 ± 67 Pa for Fib5-Silk 0.5%, and up to 456 ± 32 Pa for Fib5-Silk 1%. Additionally, the G′ of the treated scaffolds (Figure 4b-red columns) was significantly higher than for the non-treated counterparts (Figure 4b-blue columns) for Fib5-Silk 0.5% and for Fib5-Silk 1% (*p* < 0.001). Similarly, the ethanol treatment also elicited an increase in the G′′ values of scaffolds Fib5-Silk 0.5% and Fib5-Silk 1% (Figure 4c).

To determine if the scaffolds behave in a more elastic way than in a viscous way, or *vice versa*, the elastic moduli values (G′) were compared with the viscous ones (G′′), as represented in Table 2. The loss factor (tan delta), that is, the ratio between G′′ and G′, was lower than 0.1 for all the tested scaffolds (see Table 2). In other words, the elastic moduli were more than 10 times higher than the viscous moduli, which indicates a predominantly elastic behavior of the scaffolds.

### 3.3. Cell Adhesion of HUVECs to the Composite Scaffolds

Cell adhesion tests were performed in order to verify if the bioactivity of the fibrin was maintained while combined with the silk fibroin. In order to discern the nature of the cell attachment (i.e., specific or unspecific), two sets of scaffolds were prepared (i) pre-treated with BSA (to block further unspecific cell adhesion) and (ii) non-pre-treated with BSA. In both cases, HUVECs were seeded on top of the scaffolds with media without FCS.

**Figure 5:**
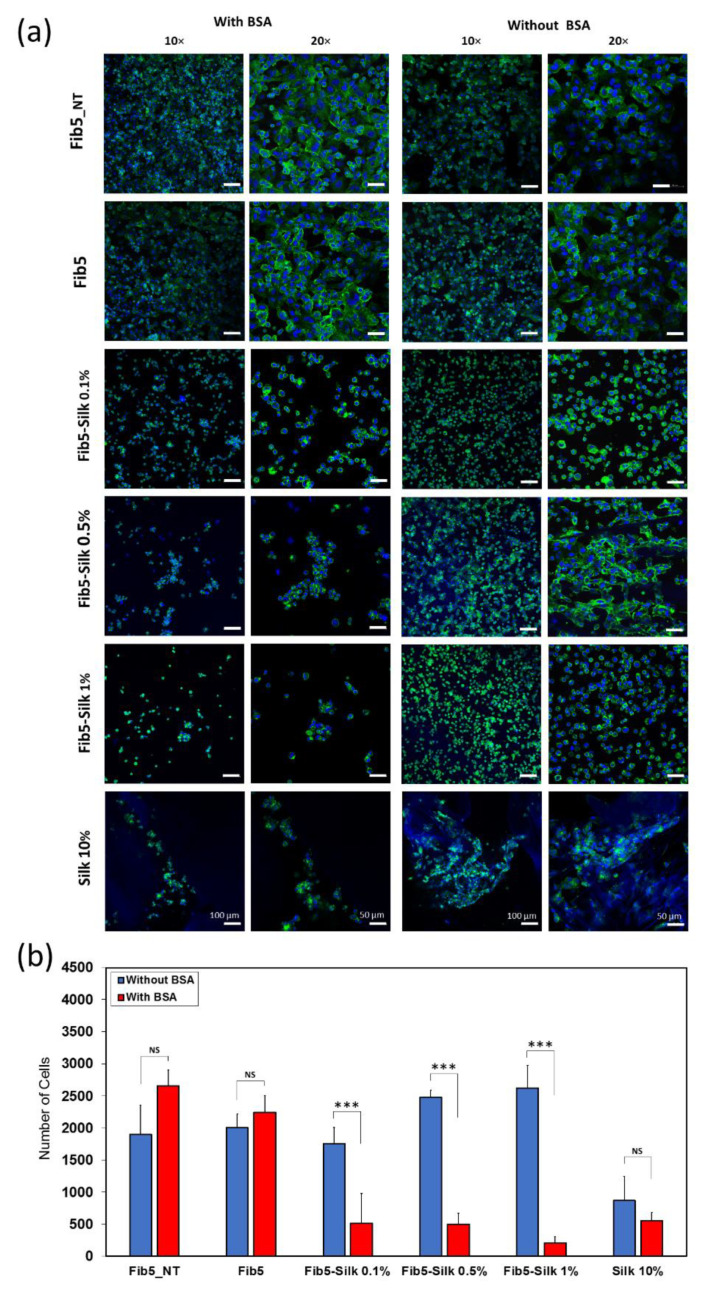
(**a**) Confocal images of the scaffolds pre-treated with bovine serum albumin (BSA) (first and second column) and without BSA pre-treatment (third and fourth columns), after 2 h in culture with HUVECs. The actin filaments in the cell cytoskeleton are stained with phalloidin (in green) and the nuclei with DAPI (in blue). (**b**) Number of HUVECs adhered in 840 μm^2^ surface of the different types of scaffolds after 2 h. Data reported as mean ± SD. For the sake of clarity only the statistical comparison between each BSA-treated scaffold with its counterpart non-treated one is included in the graph. (*** *p* < 0.001, NS: not significant).

The pure fibrin scaffolds showed a prominent number of cells even when pre-treated with BSA (Figure 5a). In contrast, the composite scaffolds (fibrin + silk fibroin) pre-treated with BSA featured a significant reduction in the number of attached cells, and specifically the composites containing the higher amount of silk fibroin (Fib5-Silk 1%) seemed to contain the lowest number of cells attached (Figure 5a). Almost no cells were observed in the scaffolds Silk 10%, and the small number of cells visualized seemed to be entrapped in the cloud-like structure of the silk fibroin.

The quantitative evaluation of these observations corroborated that the cell number decreased with an increase in the silk fibroin concentration (Figure 5b). This can be explained by the lack of the RGD motif on the sequence of silk fibroin, which is key for specific cell adhesion on the substrate. On the other hand, all the composite scaffolds non-coated with BSA succeeded in supporting HUVECs attachment (Figure 5b-blue bars), and no statistical differences were detected between fibrin and fibrin + silk fibroin under these conditions (*p* > 0.05). Regarding the morphology, the cells presented a predominantly round shape on the composite scaffolds, in contrast to the extended and cobblestone shape adopted when cultured on the fibrin scaffolds (higher magnification images in Figure 5a).

**Figure 6:**
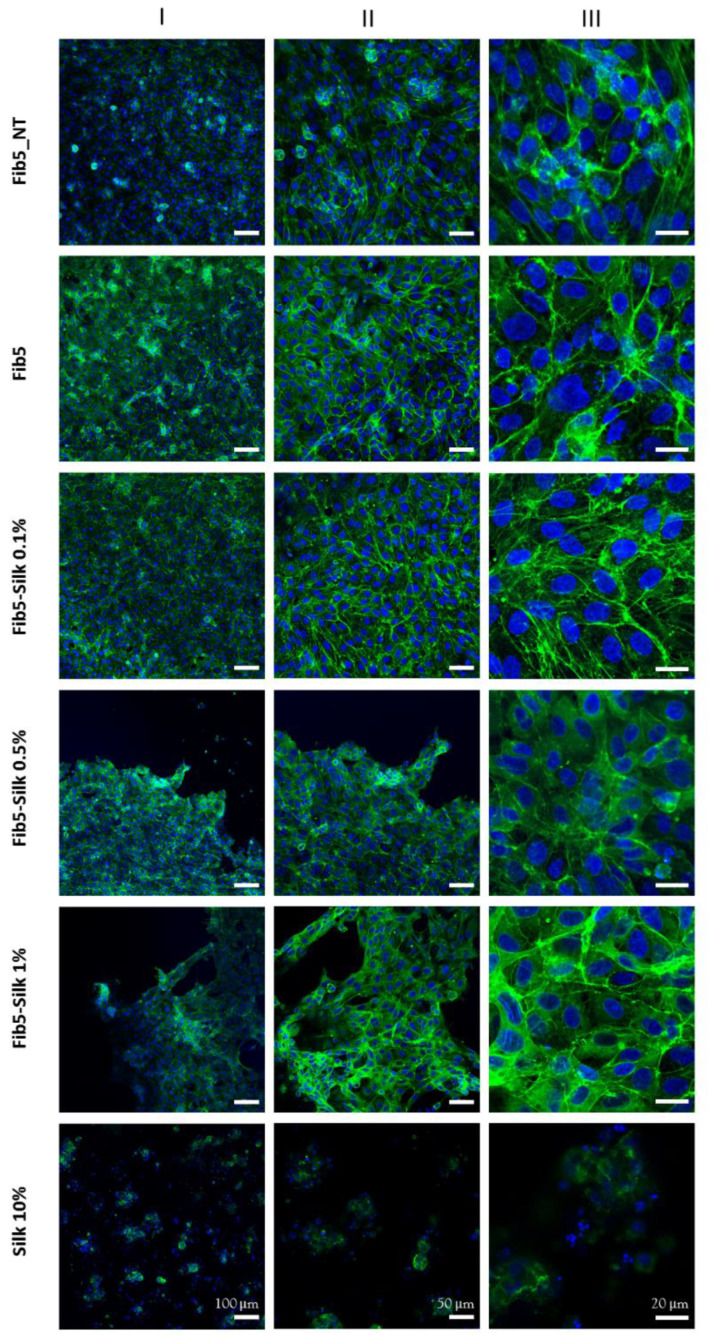
Confocal images of the scaffolds after seeding and culture with HUVECs under standard conditions. The actin filaments in the cell cytoskeleton are stained with phalloidin (in green) and the nuclei with DAPI (in blue). The left column (**I**) shows an overview, the middle column (**II**) represents the scaffolds at higher magnification, and the right column (**III**) shows a closer view of the morphology of the cells.

To check the ability of the scaffolds to support cell attachment under standard culture conditions, HUVECs were also cultured on the scaffolds for 24 h, with medium containing FCS. The cells appeared confluent and with a cobblestone shape in Fib5-Silk 0.1% samples, and less but still confluent in the Fib5-Silk 0.5% and Fib5-Silk 1% scaffolds (Figure 6). The cells displayed a spread and elongated morphology, even for the higher concentration of silk fibroin (Fib5-Silk 1%), which suggests a well cell attachment on the surface. In contrast, the scaffolds Silk 10%, after 24 h of culture, did not show any change concerning the cell shape, which was still round.

### 3.4. Evaluation of the Cell-Mediated Scaffold Contraction

**Figure 7:**
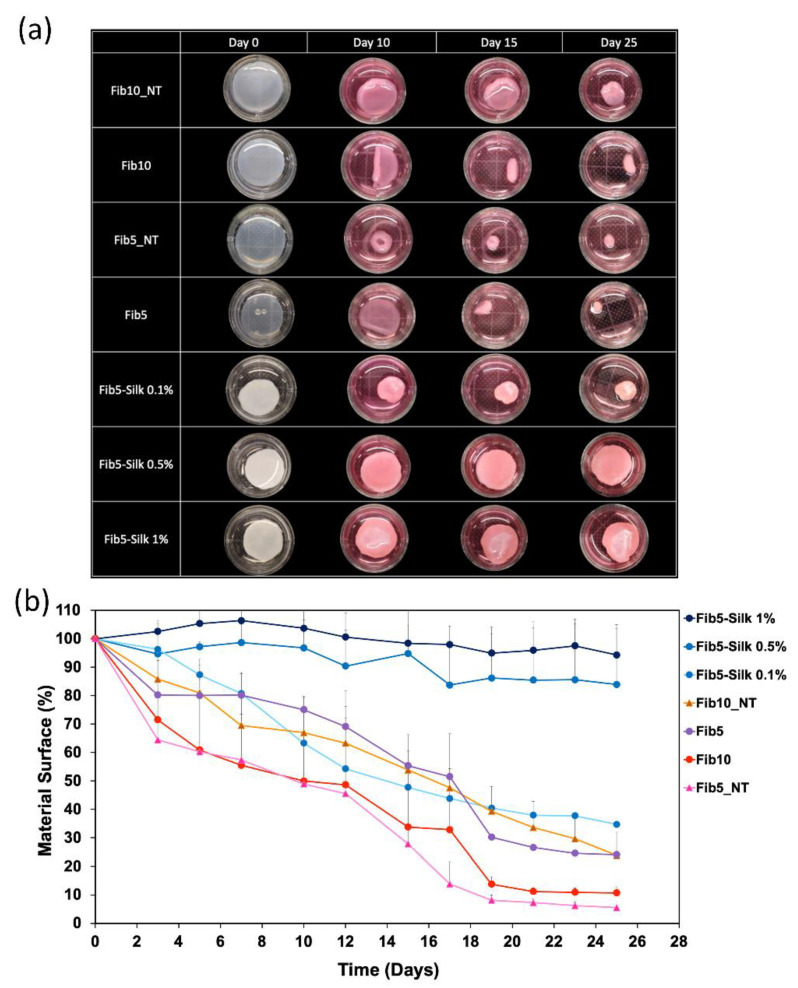
Evaluation of scaffold cell-mediated contraction over time. (**a**) Image of each scaffold before culture (time 0) and at different time points (10, 15 and 25 days) of culture with human arterial smooth muscle cells (HUASMCs). (**b**) Evaluation of cell-induced surface contraction of the fibrin/ silk fibroin scaffolds, at different time points of cell culture with HUASMCs at 37 °C and 5% CO_2_. The data are expressed as percentage of the initial surface. The data are reported as mean ± SD (*n* = 3).

After culturing with HUASMCs, Fib5_NT scaffolds experienced the highest surface contraction over time (Figure 7a), and after 27 days, their size was only 5% of their original one (Figure 7b). In other words, they exhibited a surface contraction of 94.5 ± 0.4%, in relation to their original area. Fib10_NT also experienced a prominent contraction of 77 ± 8%. On the other side, Fib5-Silk 1% and Fib5-Silk 0.5% showed a minimal surface reduction of about 5 and 15%, respectively. Fib5-Silk 0.1% was subjected to a drop in surface of 66 ± 8%.

**Figure 8:**
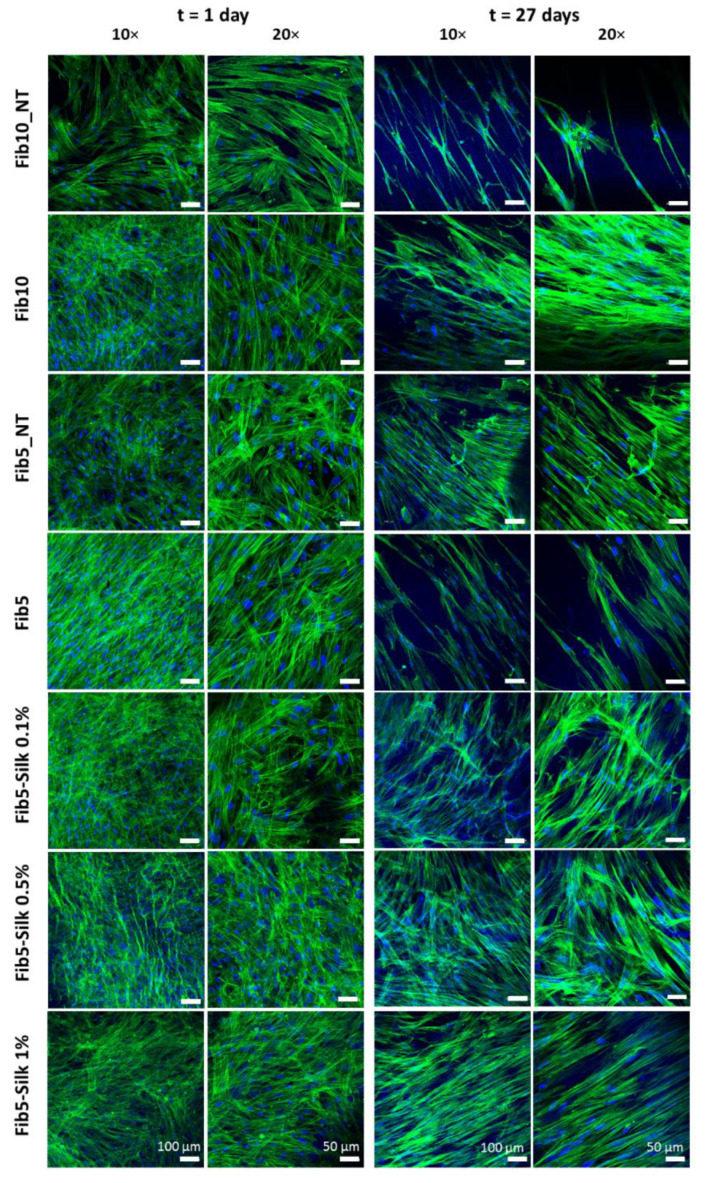
Confocal images of the scaffolds cultured with human arterial smooth muscle cells (HUASMCs) at two different time points: 1 day and 27 days. Actin filaments of the cell cytoskeleton appear in green (phalloidin staining) and nuclei in blue (DAPI staining).

To investigate if the cells were indeed attached to the composite scaffolds and they are applying their contractile force on them, the samples were stained at two different time-points, i.e., after 1 day and after the end of the culture (at day 27). Indeed, after 1 day of culture, the cells were spread and confluent in all the conditions tested (Figure 8), while at day 27, the number of cells visibly decreased in the controls (i.e., pure fibrin scaffolds), but not in the composites scaffolds. The limited adhesion of the cells to the fibrin scaffolds at this longer time-point could be related to the acute retraction, which can affect the effective support of the cells and interfere with an adequate mechano-transduction.

### 3.5. Fabrication of Composite Scaffolds with Tubular Shape

**Figure 9:**
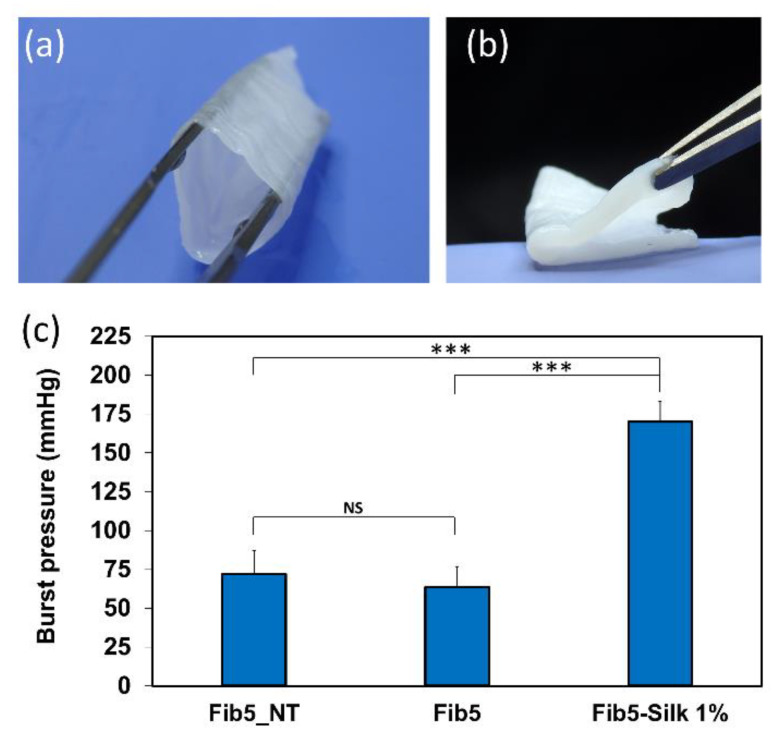
(**a**) Tubular scaffold of Fib5-Silk 1%. (**b**) Fib5-Silk 1% tubular scaffold collapsed under gravity, and further bended 180° on the circumferential axis. (**c**) Burst pressure values of the different scaffolds expressed as mean ± SD (*** *p* < 0.001; NS: not significant).

We explored the feasibility of applying the developed approach to the fabrication of composite scaffolds of a tubular shape (Figure 9a). The composite scaffolds were easy to demold in tubular form, in contrast to the controls of Fib5_NT and Fib5, which could not be demolded without breaking or deforming the tubular shape. The tubular composite scaffolds showed great handleability and outstanding flexibility, and indeed they collapsed under gravity and were able to bend 180° (Figure 9b).

The burst pressure of the composite Fib5-Silk 1% (170 ± 19 mmHg) was significantly higher (*p* < 0.001) than those displayed by the scaffolds Fib5_NT and Fib5 (72 ± 15 and 63 ± 13 mmHg, respectively).

## 4. Discussion

Single polymers often fall short in fulfilling the needed requirements for their successful application. Indeed, materials in nature are rarely used as single polymers, and are rather arranged into composites [36], showing enhanced properties when compared to the single constituents. In this way, complementary or inversely correlated properties (e.g., flexibility and strength) can be combined in the same system. Consequently, the pursuit of advanced scaffolds should focus on the selection of adequate materials as well as on the appropriate fabrication techniques to enable synergy between the different components and to match the needs of the target tissue [37].

In this study, we proposed the combination of silk fibroin (as strong, bioprocessable) and fibrin (flexible, bioactive) materials, in order to obtain composite scaffolds with advanced properties for TE, specifically in regards to resistance to cell-mediated gel contraction, bioactivity and mechanical performance. For their biofabrication, we proposed a strategy that exploits a double dual injector system to mix the fibrin and silk fibroin in a homogenous and reproducible way. Previous studies suggest that an optimal concentration of fibrin ranges between 3 and 5 mg/mL [38]. Therefore, the fibrin concentration was fixed at 5 mg/mL and the silk fibroin content varied (0.1%, 0.5%, 1% *w*/*w*) in the composites to determine which composite scaffold attained the best mechanical behavior and resistance against cell-medicated gel contraction, while still featuring an adequate cell attachment.

At both the macro and micro level, the resulting composites showed a homogenous and reproducible architecture characterized by an interconnected porous structure. The open porosity displayed by the composites was not hampered by the increase in content of the silk fibroin. The pore size on the composite scaffolds ranged between 5 μm and 15 μm of diameter, which has been reported as suitable for cell infiltration and nutrient diffusion [39,40,41].

The proposed ethanol-based protocol of crosslinking is suitable for seeding the cells on top but not within the scaffolds. However, the increase in the mechanical properties paves the way for the application of these scaffolds as cell-free systems, able to provide sufficient support and handleability for implantation without any cells or any pre-conditioning steps [42,43,44]. The biodegradable nature of both components (fibrin and silk fibroin) together with the open porosity provides a priori a suitable scenario for cell colonization and endogenous remodelling.

Regarding the rheological behavior, the formation of a gel for non-treated scaffolds proves that the silk fibroin did not obstruct the polymerization of the fibrin. The presence of silk fibroin within the composites and subsequent crosslinking (ethanol treatment) contributed greatly to increase the mechanical properties of the fibrin, from an elastic modulus of 23 ± 5 Pa for fibrin alone, to 456 ± 31 Pa for the Fib5-Silk 1%. The β-sheets formation of the silk fibroin is essential to boost the mechanical properties (Figure 4b), as the increase of content of the silk fibroin is only evident when the crosslinking is made with ethanol. The composite with the least in silk fibroin content (Fib5-Silk 0.1%), did not experience an increase in elastic modulus after ethanol treatment (Figure 4a). A possible explanation might be that the silk fibroin concentration in the composite is too low to enable the formation of a silk fibroin network that can support mechanical forces. Both the ethanol-treated and the non-treated fibrin scaffolds (Fib5 and Fib5_NT) showed elastic modulus of just a few pascals, and the ethanol treatment did not elicit an increase in the mechanical properties of the pure fibrin gels. Therefore, the increased mechanical performance observed in the composite scaffolds can be attributed to the ethanol-induced annealing of the silk fibroin contained within them.

A cell adhesion study with HUVECS was carried out in order to check whether the fibrin contained within the hybrid composites was able to provide the specific binding sites, or whether the adhesion was impaired due to the presence of silk fibroin.

Fibrin scaffolds pre-treated with BSA (blocking agent) showed effective cell adhesion, while the composites (fibrin + silk fibroin) showed that, by increasing the silk fibroin content, the number of cells attached decreased by 70% in the case of Fib5-Silk 0.1%, 80% for Fib5-Silk 0.5% and 92% for Fib5-Silk 1% by (Figure 5b). However, the number of cells attached on the composites was significantly higher than for the pure silk fibroin scaffolds. In other words, the inclusion of fibrin within the silk fibroin scaffold improves the specific adhesion of the cells, although such adhesion is reduced when compared to pure fibrin scaffolds, as expected. However, after just 24 h under standard culture conditions (no BSA pre-treatment and media supplemented with FCS), the endothelial cells (HUVECs) seemed to cover most of the composites’ surfaces and assume an elongated morphology, which indicates a good adhesion to the substrate. The endothelial cells line the lumen of the vessels and grant hemocompatibility [45]. Additionally, they play an essential role in the dynamic regulation of the vascular hemostasis (e.g., the patency of the blood vessel), by secreting different kinds of vasoregulatory factors [46]. Therefore, the consecution of a confluent and adherent endothelial layer paves the way for the application of these scaffolds in the cardiovascular TE field.

Additionally, the bioactivity of fibrin was not affected by the ethanol-treatment, i.e., regardless of the culture conditions (Figure 5 and Figure 6), Fib5 preserved the same remarkable cell-adhesion as fibrin non-treated with ethanol. This observation agrees with previous studies, in which the ethanol sterilization was shown to not significantly change the mechanical and chemical properties of the fibrin gels [47].

One of the major drawbacks of fibrin as a biomaterial is the cell-mediated retraction that the fibrin-scaffold experiences [48]. Such a retraction can hamper the consecution of implants with a defined shape over time, and therefore, preclude the applicability of fibrin as a fabrication material in TE. However, the composites resulting from blending the fibrin with silk fibroin showed not only an increased in the mechanical properties, but also a significant reduction of cell-mediated scaffold contraction. Figure 7a shows how during 27 days of culture with HUASMCs (strong contractile cells), the surface of the fibrin scaffold changes drastically, while the presence of silk fibroin clearly halts the cell-mediated contraction that the pure fibrin is subject to. The addition of Fib10_NT to the conditions demonstrated that the increase in material content (fibrin concentration) is not the leading factor in the prevention of the fibrin size reduction in culture. Indeed, the Fib10_NT, although in a lower extent than the Fib5_NT, was subjected to cell-mediated scaffold contraction of almost 80% of its original surface area (Figure 7). Moreover, this high concentration of fibrin in Fib10_NT makes the scaffold more compact and rigid [38], which can negatively impact the proliferation of cells, as shown in the confocal microscope images (Figure 8). In contrast, the addition of silk fibroin to the fibrin in the composites resulted in a confluent layer of HUASMCs.

In a recent study, fibrin and elastin-like recombinamers (ELRs) were combined [49], to obtain better mechanical and biological properties. Despite the promising approach of that study, a final concentration of ELRs of 1% (*w*/*w*) did not preserve the shape of the scaffold. While a higher content could maintain the scaffold shape, the increase of ELR concentration resulted in a dense and compact scaffold, which hampered cell proliferation [49]. In contrast, in our approach, the addition of silk fibroin (1% *w/w*) was sufficient to fully guarantee the stability of the gels and prevent cell-mediated scaffold contraction. Additionally, the HUASMCs were well distributed all over the composite surfaces, with an elongated morphology that indicates a good adhesion on the substrate. Such stability is a target property in many TE applications, including cardiovascular ones. For example, a tissue-engineered heart valve must be biofabricated with a resistance to cell-mediated scaffold contraction in order to guarantee a proper opening and closing of the implant. If the leaflets contract, the coaptation is hampered, and therefore also the functionality of the whole implant [50].

By applying the injection molding fabrication approach described in Section 2.6, we demonstrated that it was possible to obtain tubular scaffolds made of fibrin/silk fibroin, without the use of any textile support. The tubular structures are ubiquitous within the human body (e.g., arteries, veins, urine conducts, and trachea). Therefore, the feasibility to fabricate fibrin/silk fibroin composites in a tubular shape paves the way for the application of these composites in several TE applications. In addition, the composite tubular scaffolds displayed great flexibility, especially considering that the tubular scaffolds could be bended without creating any kink (Figure 9b). This flexibility is highly relevant in the cardiovascular field and is a material requisite when engineering vascular grafts and heart valves. For these applications, the material needs to withstand considerable internal pressure without bursting, therefore the burst pressure of the composites was also investigated.

The incorporation of silk fibroin into the scaffolds leaded to a significant increase in the burst strength of the fibrin scaffolds. As reported in Figure 9c, the burst pressure of the composite Fib5-Silk 1% was two times higher than the respective values of treated and non-treated pure fibrin scaffolds (i.e., Fib5 and Fib5_NT).

Future work is required to acquire the full picture regarding the possible implementation of the composite scaffolds made of fibrin/silk fibroin in the vascular tissue applications, such as testing the compliance of the composites´ tubular scaffolds or testing their strength in holding the suture lines in different directions.

## 5. Conclusions

In conclusion, it was possible to develop a scaffold made of two natural polymers fibrin and silk fibroin, that are able to offer the high mechanical properties of silk fibroin and, at the same time, provide the extraordinary bioactivity of fibrin. The simplicity of the fabrication method, the versatility and flexibility undertaking different shapes, the successful balance between the mechanical properties and the bioactivity meet the requirements for the composite scaffolds to be employed in cardiovascular TE applications.

## Figures and Tables

**Table 1:** Different conditions used for the fabrication of the scaffolds.

Nomenclature	Ethanol Bath	[Fibrin]	[Silk Fibroin]
**Fib5_NT**	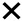	5 mg/mL	/
**Fib5**	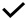	5 mg/mL	/
**Fib5-Silk 0.1%**	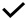	5 mg/mL	0.1%
**Fib5-Silk 0.5%**	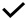	5 mg/mL	0.5%
**Fib5-Silk 1%**	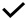	5 mg/mL	1%

**Table 2:** Values of the delta tangent (tan δ = G′′/G′) for the ethanol-treated scaffolds, expressed as mean ± SD.

Scaffold	Fib5	Fib5-Silk 0.1%	Fib5-Silk 0.5%	Fib5-Silk 1%
**tan δ**	0.078 ± 0.010	0.080 ± 0.003	0.078 ± 0.003	0.074 ± 0.007

## Data Availability

Data associated with this study is available upon request to the corresponding authors.

## References

[1] Gore P.M., Kandasubramanian B. (2018). Functionalized Aramid Fibers and Composites for Protective Applications: A Review. Ind. Eng. Chem. Res..

[2] Sell S.A., Wolfe P.S., Garg K., McCool J.M., Rodriguez I.A., Bowlin G.L. (2010). The Use of Natural Polymers in Tissue Engineering: A Focus on Electrospun Extracellular Matrix Analogues. Polymers.

[3] Swetha M., Sahithi K., Moorthi A., Srinivasan N., Ramasamy K., Selvamurugan N. (2010). Biocomposites containing natural polymers and hydroxyapatite for bone tissue engineering. Int. J. Biol. Macromol..

[4] Taylor P.M., Cass A.E.G., Yacoub M.H. (2006). Extracellular matrix scaffolds for tissue engineering heart valves. Prog. Pediatric Cardiol..

[5] Reddy M.S.B., Ponnamma D., Choudhary R., Sadasivuni K.K. (2021). A Comparative Review of Natural and Synthetic Biopolymer Composite Scaffolds. Polymers.

[6] Bhardwaj N., Sow W.T., Devi D., Ng K.W., Mandal B.B., Cho N.-J. (2014). Silk fibroin–keratin based 3D scaffolds as a dermal substitute for skin tissue engineering. Integr. Biol..

[7] Brown A.C., Barker T.H. (2014). Fibrin-based biomaterials: Modulation of macroscopic properties through rational design at the molecular level. Acta Biomater..

[8] Noori A., Ashrafi S.J., Vaez-Ghaemi R., Hatamian-Zaremi A., Webster T.J. (2017). A review of fibrin and fibrin composites for bone tissue engineering. Int. J. Nanomed..

[9] Ferguson J., Nürnberger S., Redl H., von Byern J., Grunwald I. (2010). Fibrin: The Very First Biomimetic Glue—Still a Great Tool. Biological Adhesive Systems: From Nature to Technical and Medical Application.

[10] Spicer P.P., Mikos A.G. (2010). Fibrin glue as a drug delivery system. J. Control. Release J. Control. Release Soc..

[11] Persinal-Medina M., Llames S., Chacón M., Vázquez N., Pevida M., Alcalde I., Alonso-Alonso S., Martínez-López L.M., Merayo-Lloves J., Meana Á. (2022). Polymerizable Skin Hydrogel for Full Thickness Wound Healing. Int. J. Mol. Sci..

[12] Fan Y., Perez K., Dym H. (2020). Clinical Uses of Platelet-Rich Fibrin in Oral and Maxillofacial Surgery. Dent. Clin. N. Am..

[13] Lopezcarasa-Hernandez G., Perez-Vazquez J.F., Guerrero-Naranjo J.L., Martinez-Castellanos M.A. (2021). Versatility of use of fibrin glue in wound closure and vitreo-retinal surgery. Int. J. Retin. Vitr..

[14] Vinatier C., Gauthier O., Masson M., Malard O., Moreau A., Fellah B.H., Bilban M., Spaethe R., Daculsi G., Guicheux J. (2009). Nasal chondrocytes and fibrin sealant for cartilage tissue engineering. J. Biomed. Mater. Res. Part A.

[15] Patel S., Rodriguez-Merchan E.C., Haddad F.S. (2010). The use of fibrin glue in surgery of the knee. J. Bone Jt. Surgery. Br. Vol..

[16] Moreira R., Neusser C., Kruse M., Mulderrig S., Wolf F., Spillner J., Schmitz-Rode T., Jockenhoevel S., Mela P. (2016). Tissue-Engineered Fibrin-Based Heart Valve with Bio-Inspired Textile Reinforcement. Adv. Healthc. Mater..

[17] Wolf F., Paefgen V., Winz O., Mertens M., Koch S., Gross-Weege N., Morgenroth A., Rix A., Schnoering H., Chalabi K. (2019). MR and PET-CT monitoring of tissue-engineered vascular grafts in the ovine carotid artery. Biomaterials.

[18] Ye Q., Zünd G., Benedikt P., Jockenhoevel S., Hoerstrup S.P., Sakyama S., Hubbell J.A., Turina M. (2000). Fibrin gel as a three dimensional matrix in cardiovascular tissue engineering. Eur. J. Cardio-Thorac. Surg. J. Eur. Assoc. Cardio-Thorac. Surg..

[19] Rosso M.P.O., Oyadomari A.T., Pomini K.T., Della Coletta B.B., Shindo J., Ferreira Júnior R.S., Barraviera B., Cassaro C.V., Buchaim D.V., Teixeira D.B. (2020). Photobiomodulation Therapy Associated with Heterologous Fibrin Biopolymer and Bovine Bone Matrix Helps to Reconstruct Long Bones. Biomolecules.

[20] Buchaim D.V., Cassaro C.V., Shindo J., Coletta B.B.D., Pomini K.T., Rosso M.P.O., Campos L.M.G., Ferreira R.S., Barraviera B., Buchaim R.L. (2019). Unique heterologous fibrin biopolymer with hemostatic, adhesive, sealant, scaffold and drug delivery properties: A systematic review. J. Venom. Anim. Toxins Incl. Trop. Dis..

[21] Hunt N.C., Grover L.M. (2010). Cell encapsulation using biopolymer gels for regenerative medicine. Biotechnol. Lett..

[22] Schmoekel H.G., Weber F.E., Schense J.C., Grätz K.W., Schawalder P., Hubbell J.A. (2005). Bone repair with a form of BMP-2 engineered for incorporation into fibrin cell ingrowth matrices. Biotechnol. Bioeng..

[23] Barsotti M.C., Felice F., Balbarini A., Di Stefano R. (2011). Fibrin as a scaffold for cardiac tissue engineering. Biotechnol. Appl. Biochem..

[24] Thompson W.D., Smith E.B., Stirk C.M., Marshall F.I., Stout A.J., Kocchar A. (1992). Angiogenic activity of fibrin degradation products is located in fibrin fragment E. J. Pathol..

[25] Robson S.C., Shephard E.G., Kirsch R.E. (1994). Fibrin degradation product D-dimer induces the synthesis and release of biologically active IL-1 beta, IL-6 and plasminogen activator inhibitors from monocytes in vitro. Br. J. Haematol..

[26] Jockenhoevel S., Zund G., Hoerstrup S.P., Chalabi K., Sachweh J.S., Demircan L., Messmer B.J., Turina M. (2001). Fibrin gel—Advantages of a new scaffold in cardiovascular tissue engineering. Eur. J. Cardio-Thorac. Surg. J. Eur. Assoc. Cardio-Thorac. Surg..

[27] Altman G.H., Diaz F., Jakuba C., Calabro T., Horan R.L., Chen J., Lu H., Richmond J., Kaplan D.L. (2003). Silk-based biomaterials. Biomaterials.

[28] Melke J., Midha S., Ghosh S., Ito K., Hofmann S. (2016). Silk fibroin as biomaterial for bone tissue engineering. Acta Biomater..

[29] Altman G.H., Horan R.L., Lu H.H., Moreau J., Martin I., Richmond J.C., Kaplan D.L. (2002). Silk matrix for tissue engineered anterior cruciate ligaments. Biomaterials.

[30] Samal J., Weinandy S., Weinandy A., Helmedag M., Rongen L., Hermanns-Sachweh B., Kundu S.C., Jockenhoevel S. (2015). Co-Culture of Human Endothelial Cells and Foreskin Fibroblasts on 3D Silk-Fibrin Scaffolds Supports Vascularization. Macromol. Biosci..

[31] Goczkowski M., Gobin M., Hindié M., Agniel R., Larreta-Garde V. (2019). Properties of interpenetrating polymer networks associating fibrin and silk fibroin networks obtained by a double enzymatic method. Mater. Sci. Eng. C Mater. Biol. Appl..

[32] Hasturk O., Jordan K.E., Choi J., Kaplan D.L. (2020). Enzymatically crosslinked silk and silk-gelatin hydrogels with tunable gelation kinetics, mechanical properties and bioactivity for cell culture and encapsulation. Biomaterials.

[33] Malischewski A., Moreira R., Hurtado L., Gesché V., Schmitz-Rode T., Jockenhoevel S., Mela P. (2017). Umbilical cord as human cell source for mitral valve tissue engineering—Venous vs. arterial cells. Biomed. Technik. Biomed. Eng..

[34] Moreira R., Velz T., Alves N., Gesche V.N., Malischewski A., Schmitz-Rode T., Frese J., Jockenhoevel S., Mela P. (2015). Tissue-engineered heart valve with a tubular leaflet design for minimally invasive transcatheter implantation. Tissue Eng. Part C Methods.

[35] Humphries M.J., Streuli C.H., Grant M.E. (2000). Cell Adhesion Assays. Extracellular Matrix Protocols.

[36] Safinsha S., Mubarak Ali M. (2020). Composite scaffolds in tissue engineering. Mater. Today Proc..

[37] Turnbull G., Clarke J., Picard F., Riches P., Jia L., Han F., Li B., Shu W. (2018). 3D bioactive composite scaffolds for bone tissue engineering. Bioact. Mater..

[38] de la Puente P., Ludeña D. (2014). Cell culture in autologous fibrin scaffolds for applications in tissue engineering. Exp. Cell Res..

[39] Kang X., Xie Y., Powell H.M., James Lee L., Belury M.A., Lannutti J.J., Kniss D.A. (2007). Adipogenesis of murine embryonic stem cells in a three-dimensional culture system using electrospun polymer scaffolds. Biomaterials.

[40] Rnjak-Kovacina J., Wise S.G., Li Z., Maitz P.K.M., Young C.J., Wang Y., Weiss A.S. (2011). Tailoring the porosity and pore size of electrospun synthetic human elastin scaffolds for dermal tissue engineering. Biomaterials.

[41] Takahashi Y., Tabata Y. (2004). Effect of the fiber diameter and porosity of non-woven PET fabrics on the osteogenic differentiation of mesenchymal stem cells. J. Biomater. Sci. Polym. Ed..

[42] Kwan H., Chisari E., Khan W.S. (2020). Cell-Free Scaffolds as a Monotherapy for Focal Chondral Knee Defects. Materials.

[43] Wang S.-J., Jiang D., Zhang Z.-Z., Chen Y.-R., Yang Z.-D., Zhang J.-Y., Shi J., Wang X., Yu J.-K. (2019). Biomimetic Nanosilica–Collagen Scaffolds for In Situ Bone Regeneration: Toward a Cell-Free, One-Step Surgery. Adv. Mater..

[44] Wissing T.B., Bonito V., Bouten C.V.C., Smits A. (2017). Biomaterial-driven in situ cardiovascular tissue engineering-a multi-disciplinary perspective. NPJ Regen. Med..

[45] Sumpio B.E., Timothy Riley J., Dardik A. (2002). Cells in focus: Endothelial cell. Int. J. Biochem. Cell Biol..

[46] Pang J.H., Farhatnia Y., Godarzi F., Tan A., Rajadas J., Cousins B.G., Seifalian A.M. (2015). In situ Endothelialization: Bioengineering Considerations to Translation. Small.

[47] Grasman J.M., O’Brien M.P., Ackerman K., Gagnon K.A., Wong G.M., Pins G.D. (2016). The Effect of Sterilization Methods on the Structural and Chemical Properties of Fibrin Microthread Scaffolds. Macromol. Biosci..

[48] Montero A., Acosta S., Hernández R., Elvira C., Jorcano J.L., Velasco D. (2021). Contraction of fibrin-derived matrices and its implications for in vitro human skin bioengineering. J. Biomed. Mater. Res. Part A.

[49] Stojic M., Ródenas-Rochina J., López-Donaire M.L., González de Torre I., González Pérez M., Rodríguez-Cabello J.C., Vojtová L., Jorcano J.L., Velasco D. (2021). Elastin-Plasma Hybrid Hydrogels for Skin Tissue Engineering. Polymers.

[50] He S., Fontaine A.A., Schwammenthal E., Yoganathan A.P., Levine R.A. (1997). Integrated mechanism for functional mitral regurgitation: Leaflet restriction versus coapting force: In vitro studies. Circulation.

